# An In‐Depth Evaluation of CD15 Expression in Thyroid Carcinoma: A Comprehensive Systematic Review

**DOI:** 10.1002/cnr2.70519

**Published:** 2026-03-13

**Authors:** Amir Ebrahimzadeh Pasha, Amirhossein Ehsani, Sara Ashtari, Mohammad Sadeq Najafi, Bardia Baik, Mohammad Mahdi Mehrabi

**Affiliations:** ^1^ Faculty of Medicine Iran University of Medical Sciences Tehran Iran; ^2^ School of Medicine Tehran University of Medical Sciences Tehran Iran; ^3^ Faculty of Medicine Shahed University Tehran Iran

**Keywords:** CD15, papillary thyroid carcinoma, systematic review, thyroid carcinoma biomarker

## Abstract

**Background:**

Cluster of differentiation 15 (CD15) is a carbohydrate epitope implicated in cell adhesion, migration, and tumor progression. It is variably expressed in thyroid carcinoma (TC) subtypes. However, its diagnostic and prognostic value remains uncertain due to methodological heterogeneity and inconsistent findings.

**Aim:**

To systematically review the prevalence, clinicopathologic associations, and diagnostic utility of CD15 expression across TC subtypes.

**Methods and Results:**

Following PRISMA guidelines, a systematic search was conducted in May 2025 across PubMed, Scopus, and Web of Science. Sixteen observational studies evaluating CD15 expression in human TC were included. Risk of bias was assessed using the ROBINS‐I tool. A total of 1665 TC cases were analyzed. Classic papillary thyroid carcinoma (CPTC) showed moderate overall prevalence (0%–100%), follicular variant papillary thyroid carcinoma (FVPTC) demonstrated lower expression (17.6%–80%), while tall‐cell papillary thyroid carcinoma (TCPTC) exhibited the highest positivity (71%–100%). Follicular thyroid carcinoma (FTC) showed modest expression, whereas medullary thyroid carcinoma (MTC) and anaplastic thyroid carcinoma (ATC) demonstrated overall low positivity. CD15 showed high specificity (90%–100%) for PTC, but sensitivity was inconsistent, particularly in follicular variants.

**Conclusion:**

CD15 demonstrates high specificity for PTC. However, its limited sensitivity and variable expression in follicular‐patterned lesions make its prognostic value unclear. CD15 should not be used as a standalone marker; instead, it may improve diagnostic accuracy when combined with other markers in multiparametric immunohistochemical panels.

**Abbreviations**
ATC: anaplastic thyroid carcinoma
CD15: cluster of differentiation 15
CK: cytokeratin
CNB: core‐needle biopsy
CPTC: classic papillary thyroid carcinoma
CTLA4: cytotoxic t‐lymphocyte–associated protein 4
FFPE: formalin‐fixed paraffin‐embedded
FNA: fine‐needle aspiration
FTC: follicular thyroid carcinoma
FVPTC: follicular variant papillary thyroid carcinoma
GAL‐3: galectin‐3
HBME‐1: human bone marrow endothelial‐1
HR: hazard ratio
IHC: immunohistochemistry
MTC: medullary thyroid carcinoma
NR: not reported
OR: odds ratio
PD‐1: programmed cell death protein 1
PD‐L1: programmed cell death ligand 1
PFS: progression‐free survival
PICOS: population‐intervention‐comparator‐outcome‐study design framework
PRISMA: preferred reporting items for systematic reviews and meta‐analyses
PTC: papillary thyroid carcinoma
ROBINS‐I: risk of bias in non‐randomized studies of interventions
SSEA‐1: stage‐specific embryonic antigen‐1
TC: thyroid carcinoma
TCPTC: tall‐cell papillary thyroid carcinoma
WOS: web of science

## 1 Introduction

Thyroid carcinoma (TC) is the most common endocrine malignancy globally, with a rising incidence attributed to improved imaging and diagnostic techniques [1, 2]. There are four major subtypes of TC, including papillary thyroid carcinoma (PTC), which constitutes about 75%–85% of cases, followed by follicular thyroid carcinoma (FTC), medullary thyroid carcinoma (MTC), and the aggressive anaplastic thyroid carcinoma (ATC) [3, 4]. PTC has several subtypes, of which the most common are classic PTC (CPTC), follicular variant PTC (FVPTC), and tall‐cell PTC (TCPTC) [5]. While PTC and FTC typically have an excellent prognosis, ATC is linked to poor outcomes and high mortality rates [6, 7].

Accurately distinguishing between benign and malignant thyroid lesions continues to be a significant challenge in endocrine pathology [8]. Despite advancements in fine‐needle aspiration (FNA), molecular testing, and immunohistochemistry (IHC), some thyroid nodules still produce indeterminate results. This can lead to unnecessary surgeries or delayed treatments [9, 10, 11]. Therefore, there is a need for reliable immunohistochemical biomarkers that can enhance diagnostic precision and improve risk stratification.

The cluster of differentiation (CD) system classifies surface molecules on immune cells and other tissues and is identified using monoclonal antibodies [12]. These markers are crucial for understanding the types of immune cells, their signaling pathways, and their interactions in both healthy and diseased states [13]. The application of CD markers in immunohistochemistry (IHC) has become crucial for the diagnosis and classification of tumors [14]. CD15, also known as Lewis X or SSEA‐1, is a carbohydrate antigen commonly expressed on neutrophils and certain epithelial cells. It mediates cell adhesion by interacting with selectins and is implicated in leukocyte migration and possibly tumor metastasis [15, 16]. CD15 is also recognized as a marker in Hodgkin lymphoma and is found in some solid tumors, including those of the thyroid [16, 17, 18].

Numerous studies have demonstrated that benign thyroid tissues, such as nodular hyperplasia and follicular adenomas, are typically negative for CD15, whereas malignant lesions show variable expression [19]. Xu et al. found different expression in TC subtypes and none in benign tissues [20]. Oh et al. reported variable CD15 positivity across PTC subtypes, but none in adjacent benign thyroid tissue [19]. Moreover, other studies reported moderate to low expression in benign thyroid lesions, and also no expression in normal thyroid tissue was noted [21, 22]. The use of CD15 in IHC panels has been shown to improve diagnostic accuracy in differentiating malignant from benign thyroid lesions [23]. Some evidence suggests that CD15 expression may be associated with aggressive clinicopathologic features or altered recurrence risk; however, findings remain conflicting [16].

Despite the accumulating data, the practical use of CD15 in the diagnosis and prognosis of TC has long been in question. There has been a lack of standardization in expression rates, differences in monoclonal antibodies, cut‐off values, and study design, which have hindered the application of these results in a practical setting. To date, there has been no comprehensive systematic review of all major types of TC.

This systematic review seeks to examine and compare the expression of CD15 in human TC, including PTC and its major variants, FTC, MTC, and ATC. This review aims to clarify the role of CD15 in diagnostics, explore its potential significance in prognosis, and identify areas that need further investigation. The objective of this systematic review is to enhance the management of individuals diagnosed with TC.

## 2 Materials and Methods

### 2.1 Study Design

**FIGURE 1:**
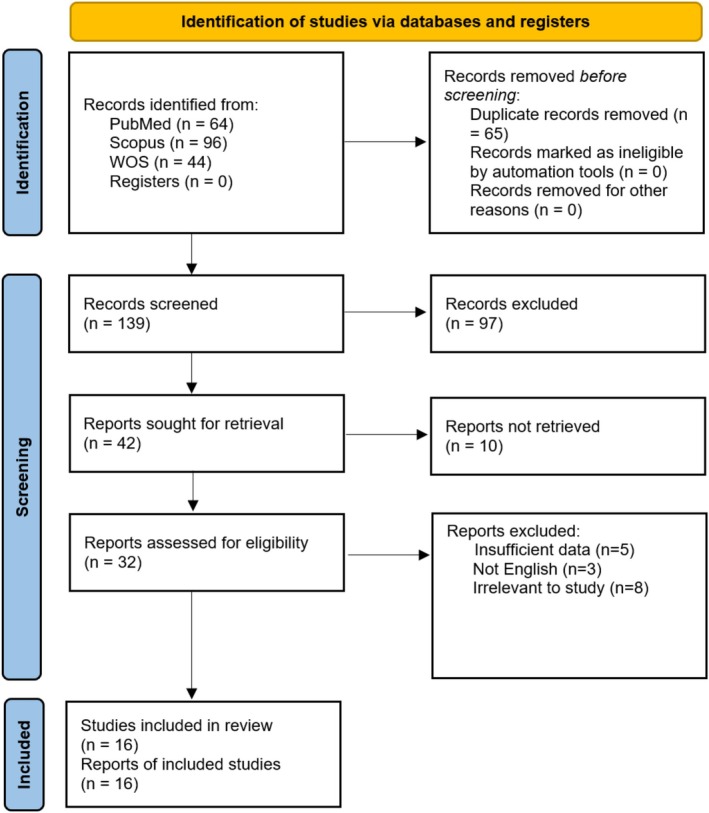
PRISMA flow diagram. The flowchart demonstrates the process of selecting studies.

The study protocol followed the guidelines set by the Preferred Reporting Items for Systematic Reviews and Meta‐Analyses (PRISMA) 2020 and was registered with PROSPERO under the code CRD420251113865 [24]. Figure 1 illustrates the PRISMA flow diagram detailing the study selection process.

### 2.2 Literature Search Strategy

A comprehensive literature search was conducted across three major databases: PubMed, Scopus, and Web of Science (WOS). Articles published up to 20th May 2025 were deemed eligible for inclusion. No date restriction was applied. The search strategy utilized a combination of Medical Subject Headings (MeSH) and free‐text terms: (“CD 15” or “CD15” or “Lewis X Antigen” [Mesh] or “Lewis X antigen” or “Lex” or “SSEA‐1 Antigen” or “SSEA‐1” or “Lex antigen” or “3‐fucosyl‐N‐acetyl‐lactosamine”) and (“Thyroid Gland” [Mesh] or “Thyroid Neoplasms” [Mesh] or “thyroid”) and (“Neoplasms” [Mesh] or “cancer” or “malignancy” or “carcinoma” or “neoplasm” or “tumor”).

Additional studies were sought by reviewing the reference lists of included articles and searching Google Scholar for relevant publications. Grey literature was screened and excluded unless it provided significant results.

### 2.3 Eligibility Criteria

Inclusion of studies was based on several predefined criteria. Studies were considered eligible if they investigated the expression of CD15 in human TC tissue. Only original, peer‐reviewed research articles were included, which could be cohort, case–control, or cross‐sectional studies. Furthermore, studies were required to provide data on the prevalence of CD15 expression, staining characteristics, or its associations with clinicopathological parameters. Only studies published in English were considered for inclusion in the final analysis.

Studies were excluded from the review if they were reviews, editorials, case reports, conference abstracts, or animal/in vitro studies. Additionally, studies that did not include data on CD15 detection in TC or research focusing exclusively on non‐thyroid malignancies or cell lines were excluded. Studies that lacked extractable data on CD15 expression or did not provide key methodological details, usable clinicopathological data, or full‐text availability were also excluded from the analysis.

### 2.4 Study Selection

PubMed (*n* = 64), Scopus (*n* = 96), and WOS (*n* = 44) identified 204 records. After removing duplicates, 139 records remained for screening. Two reviewers (M.M.M. and S.A.) independently screened the titles and abstracts for relevance. Among these, 42 articles were selected for further evaluation based on the eligibility criteria. In the second review of title and abstract screening, 10 articles were not retrieved for full‐text screening. After a full‐text review of 32 articles, 16 studies met the inclusion criteria and were included in the final analysis. Three articles were not published in English, eight were not relevant to the review topic, and five lacked sufficient data on CD15 expression in TC. Any disagreements were resolved by consensus or through consultation with a third reviewer (A.E.P.). Backward and forward citation searching were performed for any other relevant studies.

### 2.5 Data Extraction

Data extraction was performed independently by two reviewers (B.B. and M.S.N.) using a standardized form that was informed by the PICOS framework. The following key information was extracted from each of the included studies: DOI, title of article, the first author's name, the year of publication, and the country of origin. Additionally, the study design and sample size were noted for each study. If available, patient demographic information was also extracted. Information on the specific TC subtype(s) under investigation and their corresponding histopathological classification was recorded.

Regarding CD15 assessment, data were extracted on the methodology used, including details of the IHC protocol, the specific antibody and clone used, and the definition of the positivity cut‐off. Prevalence data and patterns of CD15 expression were documented, including the percentage of positive cases and the cellular localization of CD15 expression. The criteria used to define CD15 positivity, such as the intensity of staining or the percentage of stained cells, were also extracted from the studies.

Finally, information on any associations between CD15 expression and clinicopathological parameters, such as recurrence or survival, was recorded. Key statistical findings were extracted, including reported *p*‐values or diagnostic metrics, to help assess the performance of CD15 as a diagnostic or prognostic marker in TC. In cases of discrepancies in data extraction, consensus was reached through discussion between the reviewers or with the involvement of a third reviewer (M.M.M.).

### 2.6 Risk of Bias

Risk of bias was assessed independently by two reviewers (B.B. and A.E.). For non‐randomized studies, we used the Risk of Bias in Non‐randomized Studies of Interventions (ROBINS‐I) tool to evaluate bias due to confounding, selection of participants, classification of exposures, deviations from intended exposures, missing data, measurement of outcomes, and selective reporting [25]. Study‐level judgments were recorded, and disagreements were resolved by discussion or third‐party arbitration (A.E.P).

### 2.7 Data Synthesis

Given the variability in staining protocols, scoring systems, and reported outcomes, meta‐analysis was not feasible. Therefore, data were synthesized qualitatively. For descriptive comparison across histologic subtypes, we calculated the unweighted arithmetic mean of reported positivity rates from individual studies. No formal meta‐analysis or weighted pooling was performed due to heterogeneity in methodology and reporting.

## 3 Results

### 3.1 Study Selection and Characteristics

Sixteen observational studies published between 1992 and 2025, involving diverse populations across Canada, the USA, Europe, and Asia, were included in this review. The studies examined CD15 expression in various TC subtypes, mainly PTC, but also including FTC, MTC, and ATC. All studies were retrospective observational. Sample sizes varied widely, from 21 to 423 cases, with a broad range of reported ages and a higher number of female patients. Detailed characteristics of the included studies and demographic data are summarized in Table 1.

**TABLE 1:** Characteristics of included studies.

Author (Publication year)	Country	Histopathologic type (*n*)	Total sample (*n*)	Age summary (mean or median)
Mai et al. (2000) [26]	Canada	CPTC: 30 FVPTC: 25 FTC: 5	60	PTC: 33 years FTC: 39 years
Miettinen et al. (1996) [22]	USA/Finland	PTC: 138 FTC: 19 MTC: 7 ATC: 11	175	NR
Neuhold et al. (1992) [27]	Austria	MTC	47	50 years
Oh et al. (2020) [19]	South Korea	CPTC: 385 FVPTC: 17 TCPTC: 21	423	45.1 years
Ohta et al. (2015) [28]	Japan	CPTC: 19 FVPTC: 13	32	CPTC: 61.5 ± 16.7, FVPTC: 59.8
Carbajo et al. (2025) [29]	Spain	CPTC: 26 TCPTC: 8	34	CPTC: 45.3 TCPTC: 55.5
Van Hoeven et al. (1998) [30]	USA	PTC	21	NR
Xu et al. (2016) [20]	USA	PTC: 28 FVPTC: 29 FTC: 28 ATC: 10	95	NR
Fonseca et al. (1996) [21]	Portugal	PTC: 32 FTC: 12	44	NR
Alves et al. (1999) [31]	Portugal	PTC	32	NR
Bryne et al. (1994) [32]	Norway	PTC: 30 FTC: 25 ATC: 15	76	NR
Alves et al. (1999) [33]	Portugal	PTC	26	42.4 (16 cases), 49.4 (10 cases)
Längle et al. (1994) [34]	Austria	MTC (75 sporadic and 10 hereditary)	85	52 years
Hashimoto et al. (2011) [35]	Japan	PTC: 57 FTC: 22	79	PTC: 55.1 FTC: 56.6
Kim et al. (2019) [23]	South Korea	PTC	386	46.7
Koo et al. (2010) [36]	South Korea	CPTC	50	45.2

Abbreviations: ATC, anaplastic thyroid carcinoma; CPTC, classic papillary thyroid carcinoma; FTC, follicular thyroid carcinoma; FVPTC, follicular variant papillary thyroid carcinoma; MTC, medullary thyroid carcinoma; NR, not reported; TCPTC, tall‐cell papillary thyroid carcinoma.

### 3.2 Assay Methodology and Scoring Heterogeneity

The methodology and IHC staining details of the included studies are summarized in Table 2. The primary detection method for CD15 expression was IHC performed on formalin‐fixed paraffin‐embedded (FFPE) tissues from surgically resected thyroid and FNA. Multiple antibodies (with clones) and dilutions were utilized. Positivity thresholds exhibited significant variation, ranging from any detectable staining to stringent criteria that involved percentages of stained cells, which varied from 0% to 15%. Staining patterns typically included membranous and cytoplasmic localization, with notable apical/luminal accentuation in certain PTC cases.

**TABLE 2:** Summary of methodology for CD15 detection.

First Author (Year)	Antibody (Clone)	Dilution	Staining pattern	Positivity cut‐off	Specimen
Mai et al. (2000)	CD15 monoclonal	1:100	Not described separately	> 10% positive cells	FFPE surgical
Miettinen et al. (1996)	LeuM1 monoclonal	1:40–1:50	Luminal, lateral membrane, cytoplasmic	> 10% positive cells	FFPE surgical
Neuhold et al. (1992)	Leu‐M1 monoclonal	1:10	Diffuse cytoplasmic or membranous; focal stromal	> 0% positive cells	FFPE surgical
Oh et al. (2020)	Leu‐M1 monoclonal (Dako)	1:100	Cytoplasmic	> 5% positive cells	FFPE total thyroidectomy
Ohta et al. (2015)	CD15 monoclonal (Carb‐3)	1:25 (cytology), 1:50 (histology)	Focal, moderate to strong, membranous, cytoplasmic	At least one epithelial cell positive	Cytology: imprint smears; Histology: FFPE resected thyroid
Carbajo et al. (2025)	CD15 monoclonal (MMA)	Not specified	Focal and diffuse membranous	Any Focal or diffuse staining	FFPE thyroidectomy
Van Hoeven et al. (1998)	Leu‐M1‐IgM	1:40	Cytoplasmic with membranous accentuation	> 0% positive cells	Cell block sections from FNA
Xu et al. (2016)	SSEA‐1 (MC480)	1:100	Membranous predominant, some cytoplasmic	> 0% positive cells	FFPE tissue microarray sections
Fonseca et al. (1996)	SH1‐IgG3	Undiluted	Membranous (apical) or cytoplasmic	> 5% positive cells	NR
Alves et al. (1999)	SH1	1:5	Membranous (apical) or cytoplasmic	> 5% positive cells.	FFPE surgical
Bryne et al. (1994)	SH1‐IgG	1:7	Membrane or cytoplasmic staining	> 0% cells positive	FFPE
Alves et al. (1999)	SH1 (monoclonal)	1:5	NR	> 5% positive cells	NR
Längle et al. (1994)	LeuM1 monoclonal	1:10	NR	> 15% positive cells	FFPE surgical
Hashimoto et al. (2011)	CD15 monoclonal (MMA)	1:100	Cytoplasmic and/or membranous	> 5% positive cells	FFPE surgical
Kim et al. (2019)	NR	NR	Predominantly cytoplasmic	> 10% positive cells	FFPE surgical
Koo et al. (2010)	CD15 monoclonal (C3D1)	1:50	Not applicable (CD15 negative)	> 10% positive cells	FFPE microarray surgical

Abbreviations: FFPE, formalin‐fixed paraffin‐embedded; FNA, fine‐needle aspiration; NR, not reported.

### 3.3 Prevalence of CD15 Expression by Histologic Subtype

A comprehensive summary of CD15 positivity across histologic subtypes is presented in Table 3. CD15 expression varied considerably across TC subtypes. CPTC showed a moderate overall prevalence (~63%) with a wide interstudy range (0%–100%), reflecting substantial heterogeneity. FVPTC demonstrated lower and more consistent expression (~37%, range 17.6%–80%), while TCPTC showed the highest positivity (~79%, range 71%–100%) but was based on a small series. PTC (not specified) had an intermediate prevalence (~52%, range 27%–99%). Among non‐PTC subtypes, FTC exhibited modest expression (~34%, range 0%–100%), whereas MTC and ATC showed similarly low overall positivity (~22% each), with ranges from complete negativity to marked focal expression.

**TABLE 3:** CD15 positivity in thyroid carcinomas by histologic subtype.

Tumor type (*N*%)	Mai et al.	Miettinen et al.	Oh et al.	Ohta et al. (Cytology)	Ohta et al. (Histology)	Van Hoeven et al.	Xu et al.	Fonseca et al.	Bryne et al.	Hashimoto et al.	Kim et al.	Carbajo et al.	Längle et al.	Neuhold et al.	Koo et al.	Total (%)
CPTC	25/30 (83.3)	—	260/385 (67.5)	19/19 (100)	17/19 (89.5)	—	—	—	—	—	—	11/26 (42.3)	—	—	0/50 (0.0)	62.8
FVPTC	20/25 (80.0)	—	3/17 (17.6)	3/13 (23.1)	4/13 (30.8)	—	6/29 (20.7)	—	—	—	—	—	—	—	—	37.1
TCPTC	—	—	15/21 (71.4)	—	—	—	—	—	—	—	—	8/8 (100)	—	—	—	79.3
PTC (not specified)	—	136/138 (98.6)	—	—	—	15/21 (71.4)	17/28 (60.7)	30/32 (93.8)	10/30 (33.3)	43/57 (75.4)	106/386 (27.5)	—	—	—	—	51.6
FTC	5/5 (100)	13/19 (68.4)	—	—	—	—	9/28 (32.1)	7/12 (58.3)	0/25 (0.0)	4/22 (18.2)	—	—	—	—	—	34.2
MTC	—	3/7 (42.9)	—	—	—	—	—	—	—	—	—	—	11/85 (12.9)	17/47 (36.2)	—	22.3
ATC	—	0/11 (0.0)	—	—	—	—	8/10 (80.0)	—	0/15 (0.0)	—	—	—	—	—	—	22.2

Abbreviations: ATC, anaplastic thyroid carcinoma; CPTC, classic papillary thyroid carcinoma; FTC, follicular thyroid carcinoma; FVPTC, follicular variant papillary thyroid carcinoma; MTC, medullary thyroid carcinoma; TCPTC, tall‐cell papillary thyroid carcinoma.

Overall, CD15 expression was most frequently observed in papillary‐pattern tumors (especially TCPTC and CPTC) while non‐PTC subtypes generally showed lower prevalence. However, wide interstudy variability, small sample sizes, and methodological differences limit the interpretability of these estimates.

### 3.4 Association With Clinicopathological Features and Diagnostic Utility

Several studies have reported diagnostic performance metrics for CD15, showing a consistent pattern of high specificity but varying sensitivity, which depends on the tumor subtype and specimen type. For example, Mai et al. found a sensitivity of 95% and specificity of 90% using surgical FFPE samples. Ohta et al. reported very different results for cytology: the sensitivity for CPTC was 100%, while for FVPTC, it was only 23%. When examining histology, the sensitivity was 89% for CPTC and 31% for FVPTC, with a specificity of 100%. Van Hoeven et al. found a sensitivity of 71% and specificity of 95% for FNA samples, while Hashimoto et al. reported a sensitivity of 75.4% and specificity of 91.2%. Overall, these findings indicate that while CD15 has strong confirmatory value for classic and tall‐cell subtypes of PTC, its sensitivity for follicular‐variant tumors is poor.

Prognostic associations vary: some studies linked higher CD15 expression to recurrence or poorer survival, while others noted decreased recurrence or different results. The summarized clinicopathological associations and diagnostic utility metrics are provided in Table 4.

**TABLE 4:** Association of CD15 expression with clinicopathological features and diagnostic utility.

First author (Year)	Features investigated	Key findings	Association with other markers	Staining compartment
Mai et al. (2000)	Sensitivity, specificity	Sensitivity 95% Specificity 90%	CK, HBME, CD57	NR
Miettinen et al. (1996)	Benign vs. malignant	High sensitivity in PTC	HBME‐1	Membranous, cytoplasmic
Neuhold et al. (1992)	Recurrence, survival	CD15 > 15% with recurrence (*p* < 0.004)	NR	Cytoplasmic or membranous
Oh et al. (2020)	Recurrence	Positivity associated with Lower recurrence (HR 0.476, *p* = 0.029)	Lower TERT mutations, BRAF	Cytoplasmic
Ohta et al. (2015)	Diagnostic utility	Cytology: Sensitivity: CPTC 100%, FVPTC 23% Histology: sensitivity: CPTC 89%, FVPTC 31% Specificity 100% for cytology and histology	HBME‐1	Membranous, cytoplasmic
Carbajo et al. (2025)	PTC subtype distinction	High in TCPTC (*p* = 0.005)	CD56, BRAF	Membranous
Van Hoeven et al. (1998)	Diagnostic utility in FNA	Sensitivity 71% Specificity 95% for PTC	HBME‐1	Cytoplasmic with membranous accentuation
Xu et al. (2016)	Aggressive subtypes, survival	Poor prognosis in ATC (*p* = 0.036)	NR	Membranous, some cytoplasmic
Fonseca et al. (1996)	Malignant vs. benign	Higher in carcinomas	Sialyl Lewis x	Membranous or cytoplasmic
Alves et al. (1999)	Age, clinicopathologic	Lewis x with older age (*p* = 0.028)	Mucins, antigens	Membranous or cytoplasmic
Bryne et al. (1994)	NR	NR	NR	Membrane or cytoplasmic
Alves et al. (1999)	Prognosis, recurrence	Lewis x with bad outcomes	Mucins, antigens	NR
Längle et al. (1994)	Recurrence, survival	Score 1 (> 15% positive cells) with worse prognosis (*p* < 0.001)	NR	NR
Hashimoto et al. (2011)	Diagnostic accuracy	Sensitivity 75.4% Specificity 91.2%	34βE12, GAL‐3, CK19	Cytoplasmic and/or membranous
Kim et al. (2019)	Recurrence‐free survival, age, margin	Shorter PFS (OR: 1.92, *p* = 0.016), CD15 with older age (*p* = 0.036), and infiltrative margin (*p* = 0.002)	BRAF	Cytoplasmic
Koo et al. (2010)	Recurrence in PTC	No expression, no correlation	EMA, CK‐19, GAL‐3	Not applicable

Abbreviations: HR, hazard ratio; NR, not reported; OR, odds ratio; PFS, progression‐free survival.

### 3.5 Risk of Bias Assessment

**FIGURE 2:**
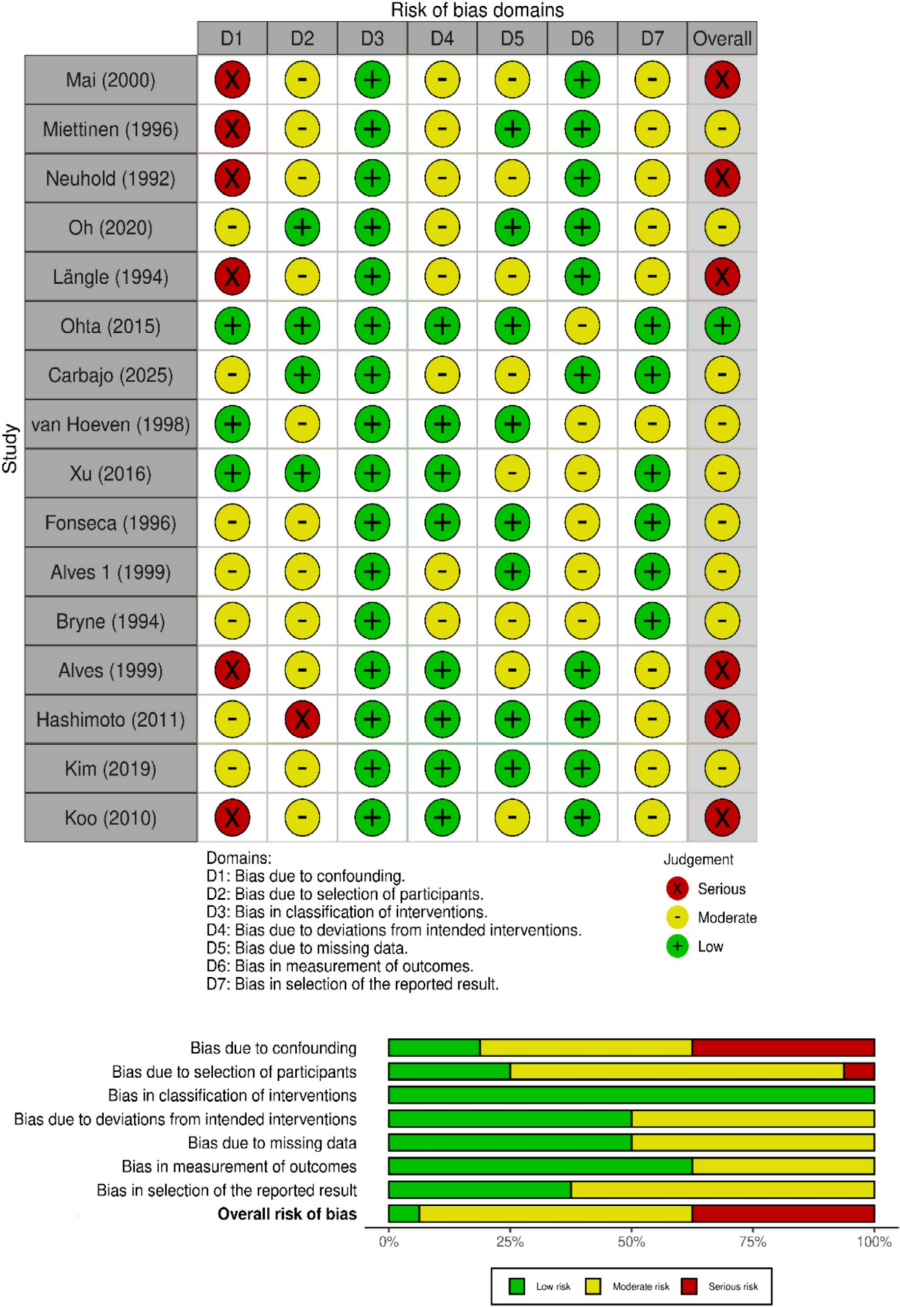
Risk‐of‐bias assessment of studies. Risk of bias was assessed for seven domains derived from the ROBINS‐I framework: D1, bias due to confounding; D2, bias due to selection of participants into the study; D3, bias in classification of interventions; D4, bias due to deviations from intended interventions; D5, bias due to missing data; D6, bias in measurement of outcomes; D7, bias in selection of the reported result. Green = low risk of bias; yellow = moderate risk of bias; red = serious risk of bias.

The qualitative assessment of the studies is summarized in Figure 2. Several concerns were identified regarding confounding (Domain 1) and participant selection (Domain 2) in the studies. Many studies either did not measure important prognostic factors or failed to report analytical approaches, such as adjustment or stratification, that could help mitigate confounding. Additionally, numerous studies lacked clear descriptions of their sampling frames or inclusion/exclusion criteria, and only a few provided protocols or prespecified analysis plans. This raises concerns about selective reporting (Domains 2 and 7).

Overall, the majority of studies were assessed to have a moderate risk of bias, though a minority exhibited a serious overall risk of bias. Consequently, the body of evidence presents a mixed risk‐of‐bias profile that must be considered when interpreting the findings of the review.

### 3.6 Publication Bias

Although we did not conduct a meta‐analysis, we performed a qualitative assessment of publication bias. Several aspects of the included evidence suggest a moderate risk of publication bias. Firstly, our review was limited to publications in English. While we screened grey literature, we excluded it unless it provided substantial results, which increases the likelihood that studies with negative or null findings were overlooked. Additionally, many of the studies included in our review were small, retrospective, and varied in their methodological rigor. Several studies did not fully report details such as antibody clones, cut‐off values, or staining criteria, which limits the transparency necessary for a comprehensive evaluation of all outcomes.

We noted a tendency in the available literature to report positive findings. Most published studies emphasized higher CD15 expression in malignant thyroid lesions, particularly in PTC, and frequently highlighted statistically significant associations or favorable diagnostic metrics, such as high sensitivity or specificity. Conversely, studies that reported absent, low, or non‐significant CD15 expression tended to present these results with less detail, smaller sample sizes, or incomplete methodological descriptions, which diminished their visibility. This asymmetry aligns with the well‐known tendency to publish positive results in biomarker research and may overstate the perceived diagnostic value of CD15.

These factors showed that the existing evidence may emphasize positive or confirmatory findings while neglecting negative or null results. As a result, the diagnostic and prognostic implications of CD15 reported in the literature may be exaggerated. To address this issue, we conducted extensive database searches, screened references, and ensured transparent reporting of variability and missing data.

## 4 Discussion

This systematic review comprehensively synthesizes findings from 16 observational studies that examine CD15 expression in various TC subtypes. The results reveal significant variability in CD15 expression among PTC, FTC, MTC, and ATC. This variability is influenced by factors such as the choice of antibody clone, staining protocols, and diagnostic thresholds. These findings highlight the need to standardize protocols to ensure accurate diagnostic usage.

FNA is the primary diagnostic tool for the initial evaluation of thyroid nodules because it is safe, straightforward, and highly specific (~99%). However, its sensitivity is limited (~72%). This limitation is especially for follicular‐patterned lesions, as it is difficult to determine invasion through cytological assessment [37, 38, 39]. This limitation often requires additional histological confirmation through core‐needle biopsy (CNB) or surgical excision. These methods offer essential tissue architecture needed for accurate diagnoses and further testing. CNB is particularly effective as it reduces nondiagnostic results and shows a higher sensitivity (83% compared to 72%) while maintaining high specificity [37, 38]. Surgical biopsy is crucial for definitively differentiating FTC from benign follicular adenomas, as it requires clear evidence of capsular or vascular invasion [40, 41].

The use of IHC significantly enhances diagnostic accuracy, particularly in indeterminate cytological cases (Bethesda III/IV). Panels that include well‐established markers such as HBME‐1, Galectin‐3, CK19, and CD56 are essential for distinguishing malignant from benign follicular lesions [42, 43]. HBME‐1 and Galectin‐3 provide considerable diagnostic sensitivity (85%–87% and 80%–85%, respectively) but moderate specificity due to focal positivity in some benign lesions [42]. Conversely, diffuse CK19 positivity and CD56 loss are highly indicative of PTC, dramatically enhancing specificity in combined panels [44]. This review highlights CD15 as an adjunctive marker within these established panels, particularly valuable in the context of classic and tall‐cell variants of PTC, demonstrating high sensitivity and specificity in histological assessments [26, 35]. Its utility, however, is significantly limited in cytological evaluations and FVPTC, where sensitivity markedly decreases [28]. Therefore, negative CD15 staining should not exclude malignancy without corroborative evidence from additional markers or histological confirmation.

Moreover, when compared with emerging biomarkers such as BRAF V600E, PD‐L1, Ki‐67, and p53, CD15 provides complementary prognostic and diagnostic insights [45, 46, 47, 48, 49]. For instance, BRAF V600E immunoreactivity signifies aggressive clinicopathological features and treatment implications, including potential targeted therapies [50, 51]. Similarly, PD‐L1 expression correlates with tumor aggressiveness and treatment responsiveness to immunotherapy, especially in refractory TCs [45, 46, 52, 53]. Ki‐67 and p53 staining further stratify prognosis, particularly in high‐grade malignancies such as ATC [54]. Hence, the inclusion of CD15 within a multiparametric panel alongside these markers can refine diagnostic accuracy and prognostic stratification [23, 29, 31, 35].

Research indicates that CD15 plays several different roles in the development of thyroid tumors. CD15 is a fucosylated glycan epitope whose expression increases due to glycosylation abnormalities commonly associated with TC, particularly changes in fucosylation and sialylation pathways [55, 56]. These glycans serve as selective ligands for E‐, L‐, and P‐selectins, allowing tumor cells to adhere to endothelial surfaces and supporting the metastatic behavior observed in PTC [16, 56]. Through these selectin‐mediated interactions, CD15 can also facilitate cancer cell survival in hypoxic or shear‐stress environments and promote angiogenesis [56, 57].

Beyond mechanical adhesion, CD15‐related glycans influence the tumor immune microenvironment. Overexpression of CD15 in PTC correlates with immunosuppressive checkpoint genes such as PD‐1, PD‐L1, and CTLA‐4 [19]. Additionally, CD15 is a defining marker of myeloid‐derived suppressor cells, which accumulate in aggressive and dedifferentiated thyroid tumors [58]. CD15 has also been associated with cancer stem‐like cell populations in TC, underscoring its connection to tumor progression and therapeutic resistance [20, 23]. These findings suggest that CD15 may contribute to the behavior of TC through its dual roles in glycan‐mediated adhesion and immune modulation.

While the present study has notable methodological strengths, it has some limitations, primarily due to the inherent variability among the included studies. Differences in antibody clones, dilution protocols, staining thresholds, retrospective study designs, and incomplete demographic reporting hindered quantitative synthesis and meta‐analysis. As a result, a qualitative and comparative interpretation was necessary. Future research should focus on standardizing CD15 assay protocols, especially regarding the selection of clones and scoring criteria. Additionally, there should be prospective multicenter validation of CD15 in diagnostic algorithms, along with a more in‐depth investigation into CD15's biological interactions with the molecular and immunologic pathways involved in thyroid carcinogenesis.

## 5 Conclusion

In summary, while CD15 is a highly specific biomarker for PTC (especially for the classic and tall‐cell variants), its diagnostic value is limited due to variable sensitivity and reduced expression in follicular‐variant PTC. Therefore, CD15 should not be used as a standalone diagnostic tool, as its absence does not reliably rule out malignancy. Instead, its clinical significance is best realized when used as an adjunctive “rule‐in” marker within a multiparametric immunohistochemical panel, which can enhance the identification of malignant thyroid nodules. Given the current methodological variability and conflicting prognostic data, CD15 is most effectively utilized in combination with established markers to address indeterminate cases. Additionally, future standardization of assay protocols is necessary to clarify its role in risk stratification.

## Author Contributions

A.E.P. conceptualized the research, developed the methodology, curated the data, validated the findings, and prepared the original draft of the manuscript. A.E. contributed to the conceptualization of the study, formulated the methodology, managed data curation, validated the results, and co‐authored the original draft. S.A. conducted the screening and selection of relevant studies, provided reviews and edits for clarity and coherence, and created visual representations to accompany the manuscript. M.S.N. executed the literature search, performed data extraction, and contributed to the writing through review and editing. B.B. conducted literature searches, performed quality assessments of studies, and facilitated data extraction. M.M.M. managed the project, provided oversight during the research process, validated the findings, and gave final approval for the manuscript. A.E.P. and A.E. contributed equally to this research endeavor. All authors have reviewed and approved the final version of the manuscript and agree to be accountable for all aspects of the work presented.

## Funding

The authors have nothing to report.

## Ethics Statement

The authors have nothing to report.

## Consent

The authors have nothing to report.

## Conflicts of Interest

The authors declare no conflicts of interest.

## Data Availability

Data sharing does not apply to this article as no datasets were generated or analyzed during the current study.
